# Age-related differences in the occurrence, severity, and distress of symptoms in older patients at the initiation of chemotherapy

**DOI:** 10.1186/s12877-023-04198-1

**Published:** 2023-08-09

**Authors:** Marlen Sunde Johannessen, Christine Miaskowski, Anne Grethe Kleven, Christine Seel Ritchie, Steven M. Paul, Ellen Karine Grov, Martine Hareide, Henrik Gaudernack, Inger Utne

**Affiliations:** 1 Department of Health Promotion, Faculty of Health Sciences, Oslo Metropolitan University, Pilestredet 32, 0166 Oslo, Norway; 2 School of Nursing, University of California, San Francisco, CA USA; 3 Massachusetts General Hospital, Harvard Medical School Boston, Harvard, MA USA

**Keywords:** Cancer, Chemotherapy, Older adults, Symptoms, Symptom assessment

## Abstract

**Background:**

Evaluate for differences in occurrence, severity, and distress ratings for 32 symptoms between younger older adults (YOA, < 70 years) and older adults (OA, ≥ 70 years) at initiation of chemotherapy.

**Methods:**

Patients (n = 125) were recruited prior to the initiation of chemotherapy and completed the Memorial Symptom Assessment Scale. Differences in occurrence, severity, and distress ratings were evaluated using Independent sample t-tests and Chi-square or Fisher’s exact tests.

**Results:**

On average, the older patients reported ten concurrent symptoms that equates with a moderate symptom burden. Symptoms with the highest occurrence rates were not always the most severe and/or the most distressing. Few age-related differences were found in patients’ symptom experiences. When age-related differences were identified, OA reported lower occurrence, severity, and distress ratings. Nine of the ten symptoms with highest occurrence rates were common for both age groups. For severity and distress, only half of the symptoms were common. In terms of severity and distress, all of the top ten ranked symptoms were in the moderate to severe range.

**Conclusions:**

Both YOA and OA reported a moderate symptom burden and severity and distress scores in the moderate to severe range. The symptoms with the highest occurrence rates were not always the most severe/or the most distressing. Our findings suggest that different dimensions of the symptom experience (i.e., occurrence, severity, and distress) warrant evaluation in older oncology patients.

## Background

The number of people aged 65 years and older is expected to double in the next two decades [1, 2]. In addition, by 2035, almost 60% of all new cases of cancer worldwide will occur in adults ≥ 65 years of age [2]. For many of these older adults, chemotherapy will be part of their treatment regimen. However, chemotherapy causes numerous adverse effects [3–7] and older oncology patients are at increased risk for chemotherapy-related toxicities [6, 7]. On average, oncology patients undergoing chemotherapy report ten unrelieved symptoms [8, 9].

Similar to younger adults, older adults with solid tumors may experience a broad range of physical and psychological symptoms. Few studies have evaluated for differences in various dimensions of the symptom experience (i.e., occurrence, severity, distress) between younger and older adults who were assessed prior to surgery [10] and during treatment [11–13].

In a study of patients receiving chemotherapy and/or radiation therapy [13], compared to patients < 60 years of age, older patients (≥ 60 years) reported significantly lower occurrence rates for 15 of 32 symptoms; lower severity ratings for 6 symptoms; and lower distress ratings for 14 symptoms. In another study of patients receiving chemotherapy [11], compared to younger patients (< 65 years), the occurrence rate for evening fatigue and the severity scores for morning and evening fatigue, morning energy, and sleep disturbance were lower in patients ≥ 65 years. The older patients reported significantly higher evening energy scores compared with the younger patients.

In a study of patients who were assessed in a palliative care clinic [12], compared to the younger patients (≤ 60 years), older patients (> 60 years) reported less pain and poorer appetite. In addition, in a study that evaluated for age differences in the occurrence, severity, and distress of 32 symptoms between younger (< 65 years) and older (≥ 65 years) patients prior to surgery [10], no differences were found in the total number of symptoms. However, compared to younger patients, older patients reported significantly lower occurrence rates for five symptoms (i.e., difficulty concentrating, feeling drowsy, feeling nervous, feeling sad, worrying); lower severity ratings for three symptoms (i.e., difficulty concentrating, feeling nervous, feeling sad); and lower distress ratings for five symptoms (i.e., feeling drowsy, pain, lack of energy, shortness of breath, worrying).

Findings across these four studies suggest that older oncology patients experience a lower or similar symptom burden as younger patients [10–13]. However, some of the inconsistent findings may be related to differences in the characteristics of the patient samples; the timing of the assessments; the use of different symptom assessment instruments; and the evaluation of different dimensions of the symptom experience (i.e., occurrence, severity, distress).

Of note, while two of the four studies dichotomized younger and older patients at 60 years of age [12, 13], and the other two used 65 years of age as the cutoff [10, 11], none of these studies provided detailed information on the symptom experience of only older oncology patients. The inconsistencies across these four studies may reflect that no clear age cutoff or definition of an older cancer patient exist. For example, while the World Health Organization (WHO) refers to the older population as ≥ 60 years [1], others use 65 and 70 years as cutpoints [14]. Equally important is the need to evaluate for differences in the symptom experience of only older oncology patients [13, 15].

To our knowledge, only one study evaluated for differences in 32 symptoms among four age groups (i.e., 60–64, 65–69, 70–74, ≥ 75) of older oncology patients receiving active treatment using the Memorial Symptom Assessment Scale (MSAS) [8]. On average, patients reported ten co-occurring symptoms and few age-related trends were found. Differences were found among the four age groups in occurrence rates for four symptoms (i.e., problems with sexual interest, lack of appetite, dizziness, swelling of arms or legs); in severity ratings for one symptom (i.e., difficulty swallowing); and in distress ratings for four symptoms (i.e., lack of energy, shortness of breath, feeling bloated, difficulty swallowing). As age increased, a decreasing linear trend was found for the occurrence of problems with sexual interest; the severity ratings for swallowing; and the distress ratings for lack of energy, shortness of breath, feeling bloated, and difficulty swallowing. In contrast, an increasing linear trend was found for the occurrence of lack of appetite, dizziness, and swelling of arms or legs. In addition, the severity and distress ratings were in the slight to moderate range for all four age groups. While this single study provides useful information on age-related differences in symptom occurrence, severity, and distress during treatment, additional research on the symptom experience of older oncology patients at the initiation of chemotherapy is warranted.

Therefore, the purpose of this study was to evaluate for difference in occurrence, severity, and distress ratings for 32 symptoms between younger older adults (YOA, < 70 years) and older adults (OA, ≥ 70 years) with gynecological or colorectal cancer at the initiation of chemotherapy.

## Methods

### Patients and settings

This analysis is part of a larger longitudinal study of changes in cognitive and physical function in older oncology patients receiving chemotherapy. The methods for this study are published in detail elsewhere [16]. In brief, patients were recruited from one community and two university hospitals in Norway. Inclusion criteria were: age ≥ 60 years; diagnosis of gynecological or colorectal cancer; scheduled to receive primary or adjuvant chemotherapy; had a Montreal Cognitive Assessment (MoCA) score of ≥ 23 [17]; and had a Karnofsky Performance Status (KPS) score of ≥ 60 [18]. A total of 208 patients were approached and 149 consented to participate (71.6% response rate). Of these 149 patients, one withdrew and nine were excluded because they had a low MoCA score (< 23). Of the 139 patients recruited, 125 completed the MSAS and were included in this analysis.

### Instruments

#### Demographic and clinical characteristics

Patients completed a demographic questionnaire that obtained information on age, gender, living arrangements, marital status, education, height and weight, and employment status. In addition, these patients completed the KPS scale that ranged from 40 (disabled; requires special care and assistance) to 100 (normal no complaints; no evidence of disease) [19, 20] and the Self-Administered Comorbidity Questionnaire (SCQ-16) [21]. The SCQ-16 includes 16 common medical conditions and evaluates the occurrence of, treatment for, and functional impact of each of the comorbid conditions (i.e., heart disease, arthritis). Total SCQ-16 scores range from 0 to 48. The SCQ-16 has well established validity and reliability [21].

#### MSAS

The MSAS was used to evaluate the occurrence, severity, and distress of 32 symptoms commonly associated with cancer and its treatment. Using the MSAS, patients were asked to indicate whether they had experienced each symptom in the past week (i.e., symptom occurrence). If they had experienced the symptom, they were asked to rate its severity and distress using a 0 to 10 numeric rating scale. The validity and reliability of the MSAS are well established in studies of oncology inpatients and outpatients [22].

### Study procedures

Oncologists or nurses approached patients prior to the initiation of chemotherapy to assess their interest in study participation. Then, patients were introduced to the research staff who explained the study; obtained written informed consent; and scheduled an appointment to perform the measures. The questionnaires were administered in the clinic or in the patient’s home prior to the initiation of chemotherapy. Research staff reviewed patients’ medical records for disease and treatment information.

### Statistical analyses

Data were analyzed using the Statistical Package for the Social Sciences (SPSS) version 27 [23]. While no clear age cutoff or definition of an older cancer patient exists, consistent with the guidelines from the International Society of Geriatric Oncology [24], in this study, older was defined as a person ≥ 70 years of age. To evaluate for between group differences in symptom occurrence, severity, and distress ratings, Independent sample t-tests for continuous variables and Chi-square or Fisher’s exact tests for categorical variables were used. A p-value of < 0.05 was considered statistically significant.

## Results

Of the 125 patients who completed the MSAS, 46.4% (n = 58) were YOA with a mean age of 65.4 (SD = 3.1) years and 53.6% (n = 67) were OA with a mean age 75.3 (SD = 4.9) years (Table 1).

**Table 1:** Differences in demographic and clinical characteristics between younger older adults (YOA) and older adults (OA)

Characteristics	YOA (< 70) 46.4% (n = 58)	OA (≥ 70) 53.6% (n = 67)	Statistics
	Mean (SD)	Mean (SD)	
Age (years)	65.4 (3.1)	75.3 (4.9)	t = -13.31; p < .001
Karnofsky Performance Status score	86.0 (10.4)	87.2 (11.4)	t = -0.61; p = .546
Body mass index (kg/m^2^)	25.2 (4.9)	26.7 (6.9)	t = -1.41; p = .162
Number of comorbidities	1.7 (1.6)	2.1 (1.9)	t = -1.30; p = .195
Self-administered Comorbidity Questionnaire score	3.2 (3.4)	4.1 (4.4)	t = -1.27; p = .208
Total number of symptoms	11.0 (5.4)	9.6 (5.6)	t = 1.38; p = .169
Time since cancer diagnosis (years)	1.9 (4.6)	1.0 (2.8)	t = 1.31; p = .192
Hemoglobin (g/dl)	12.5 (1.4)	12.8 (1.6)	t = -1.00; p = .320
	% (n)	% (n)	
Gender			
Females Males	91.4 (53) 8.6 (5)	95.5 (64) 4.5 (3)	FE; p = .470
Married or partnered (% yes)	42.9 (33)	57.1 (44)	FE; p = .458
Lives alone (% yes)	38.6 (22)	33.3 (22)	FE; p = .576
Currently employed (% yes)	33.3 (18)	1.6 (1)	FE; p < .001
Education			
Primary school High school College	13.0 (7) 51.9 (28) 35.2 (19)	19.0 (12) 44.4 (28) 36.5 (23)	x^2^ = 1.01; p = .603
Specific comorbidities (% yes)			
Heart disease High blood pressure Lung disease Diabetes Ulcer or stomach disease Bowel disease Kidney disease Liver disease Anemia/blood disease Headache Depression Osteoarthritis Back pain Rheumatoid arthritis Disease in connective-tissue Skin disease	10.3 (6) 24.1 (14) 6.9 (4) 5.2 (3) 3.4 (2) 10.5 (6) 1.8 (1) 0.0 (0) 0.0 (0) 3.6 (2) 12.3 (7) 38.6 (22) 35.7 (20) 1.8 (1) 10.7 (6) 9.1 (5)	15.9 (10) 45.5 (30) 14.3 (9) 9.4 (6) 10.9 (7) 9.4 (6) 1.6 (1) 3.1 (2) 6.6 (4) 12.9 (8) 8.1 (5) 43.1 (28) 29.5 (18) 4.8 (3) 3.3 (2) 4.7 (3)	FE; p = .429 FE; p = .015 FE; p = .245 FE; p = .496 FE; p = .168 FE; p = 1.000 FE; p = 1.000 FE; p = .498 FE; p = .120 FE; p = .099 FE; p = .548 FE; p = .713 FE; p = .555 FE; p = .621 FE; p = .150 FE; p = .469
Cancer diagnosis			
Gynecological Colorectal	84.5 (49) 15.5 (9)	92.5 (62) 7.5 (5)	FE; p = .169
Surgery prior to chemotherapy (% yes)	53.4 (31)	53.7 (36)	FE; p = 1.000
Metastasis (% yes)	75.0 (42)	81.3 (52)	FE; p = .506
Treated for recurrent disease (% yes)	39.7 (23)	26.9 (18)	FE; p = .181

Abbreviations: FE = Fisher’s Exact, g = grams, dl = deciliters, kg = kilograms, m^2^ = meters squared, SD = standard deviation

### Demographic and clinical characteristics

Except for age, employment status, and the occurrence of high blood pressure, no between groups differences were found in any of the demographic and clinical characteristics. Compared to the YOA, OA were less likely to be employed (i.e., 33.3% versus 1.6%, p < .001). In terms of high blood pressure, compared to the YOA group (24.1%), patients in the OA group (45.5%, p = .015) had a higher occurrence rate.

### Differences in symptoms

No differences were found between the OA (9.6 (± 5.6)) and the YOA (11.0 (± 5.4)) in the mean number of symptoms reported (p = .169). As shown in Table 2, compared to the OA group, the YOA group reported significantly higher occurrence rates for two (6.2%) of the 32 MSAS symptoms (i.e., lack of energy, nausea). For 30 symptoms, no significant between group differences were found. Compared to the OA group, the YOA group reported significantly higher severity scores for difficulty swallowing and vomiting. Compared to the OA group, the YOA group reported significantly higher distress ratings for changes in skin and vomiting.

**Table 2:** Differences in symptom occurrence, severity, and distress between younger older adults and older adults

Symptom	Occurrence			Severity					Distress				
	YOA (< 70) 46.4% (n = 58)	OA (≥ 70) 53.6% (n = 67)	p-value^a^	YOA (< 70) 46.4% (n = 58)		OA (≥ 70) 53.6% (n = 67)		p-value	YOA (< 70) 46.4% (n = 58)		OA (≥ 70) 53.6% (n = 67)		p-value
	%	%		Mean	SD	Mean	SD		Mean	SD	Mean	SD	
Lack of energy	87.7	70.3	0.026	5.0	2.4	4.5	2.2	0.301	4.2	3.2	3.5	3.1	0.273
Worrying	69.0	54.5	0.139	4.7	2.2	4.1	2.6	0.314	4.5	2.7	4.4	3.1	0.900
Pain	69.6	54.7	0.132	4.4	2.5	4.8	2.1	0.468	4.2	3.0	4.0	2.9	0.816
Feeling drowsy	59.6	59.1	1.00	4.2	1.8	4.0	2.3	0.715	2.3	2.3	2.5	3.0	0.830
Dry mouth	51.8	52.3	1.00	4.7	2.4	4.5	2.5	0.741	2.5	2.5	3.5	3.2	0.184
Difficulty sleeping	48.3	43.8	0.716	4.3	1.9	4.4	2.8	0.955	3.2	2.0	3.2	3.2	0.990
Feeling bloated	50.0	39.7	0.277	5.4	1.9	4.5	2.6	0.150	4.3	2.8	4.0	3.4	0.745
Lack of appetite	46.6	42.2	0.716	4.3	1.9	4.9	2.4	0.258	3.5	1.8	4.0	3.1	0.491
Constipation	46.6	42.2	0.716	4.8	2.7	5.3	2.5	0.493	4.4	3.4	5.0	3.0	0.513
Feeling sad	48.3	35.4	0.199	4.1	2.4	4.3	2.2	0.763	3.5	2.7	4.2	2.6	0.368
Numbness/tingling in hands/feet	34.5	42.2	0.457	4.4	2.9	4.4	2.8	0.910	2.6	3.4	3.4	3.3	0.446
Sweats	42.1	34.4	0.454	5.1	3.0	3.8	3.0	0.148	3.5	3.4	2.8	2.8	0.444
Difficulty concentrating	44.6	32.8	0.254	3.6	2.0	3.3	2.4	0.718	3.7	2.9	2.8	2.9	0.338
Weight loss	35.1	37.5	0.851	3.2	2.0	3.3	2.0	0.827	2.5	2.6	3.1	3.1	0.505
Dizziness	36.8	32.3	0.703	4.0	2.4	3.9	2.9	0.950	3.7	2.8	2.8	2.7	0.337
Shortness of breath	35.1	32.8	0.849	5.0	2.5	4.2	3.1	0.360	4.5	3.2	3.9	3.4	0.575
Feeling nervous	33.3	30.8	0.846	4.6	2.4	4.3	2.4	0.736	4.6	2.3	5.2	2.9	0.489
Nausea	42.1	21.9	0.019	3.4	2.2	3.2	2.0	0.846	3.0	2.4	3.0	2.5	0.957
Change in the way food tastes	31.0	27.0	0.690	3.9	1.6	4.5	2.3	0.392	2.2	1.9	2.9	2.4	0.360
Cough	21.1	24.6	0.672	2.7	2.2	2.6	2.1	0.937	2.3	2.8	1.2	2.2	0.287
Feeling irritable	24.1	20.0	0.664	3.4	1.9	3.8	2.9	0.661	2.2	1.8	2.9	2.5	0.428
Problems with sexual interest/activity	28.8	19.0	0.265	6.7	3.3	4.9	3.9	0.223	4.5	3.3	2.6	4.3	0.252
Diarrhea	20.7	16.9	0.648	5.3	2.9	3.5	3.1	0.188	3.9	3.9	3.1	4.7	0.682
Problems with urination	15.8	16.1	1.00	5.1	2.8	4.6	4.0	0.754	5.4	2.8	4.0	4.3	0.409
Swelling of arms or legs	8.8	19.7	0.124	6.4	4.1	4.3	2.9	0.235	5.0	5.2	4.1	3.7	0.728
Changes in skin	15.5	12.3	0.614	6.2	3.1	4.9	2.9	0.369	7.3	2.4	3.5	3.6	0.044
Mouth sores	12.3	14.1	0.796	3.6	2.1	4.8	2.7	0.354	2.6	2.2	4.8	2.8	0.124
I don´t look like myself	17.5	9.4	0.282	5.0	2.9	4.8	3.4	0.907	4.4	4.1	4.0	3.5	0.832
Itching	12.1	14.1	0.794	3.5	2.8	2.9	2.1	0.640	0.2	0.4	2.1	2.4	0.103
Difficulty swallowing	6.9	14.1	0.248	5.0	2.2	2.6	1.7	0.047	4.8	3.9	2.1	2.4	0.156
Hair loss	10.3	10.9	1.00	6.0	3.5	5.2	3.7	0.698	3.3	4.1	3.9	4.9	0.840
Vomiting	5.3	7.8	0.721	5.0	2.0	1.4	1.9	0.046	6.5	0.7	0.0	0.0	0.001

^a^Fisher´s Exact Test. p-value of < 0.05 is considered statistically significant

Symptom severity and distress scores are for those patients who reported the occurrence of the symptom

Symptom severity scores ranged from 0 (none) to 10 (intolerable)

Symptom distress scores ranged from 0 (not at all distressing) to 10 (very distressing)

Abbreviations: OA, older adult; SD, standard deviation; YOA, younger older adult

#### Comparison of highest ranked symptoms

Table 3 list the symptoms with the highest occurrence rates and severity and distress scores for the two age groups. Occurrence rates for the top ten symptoms ranged from 46.6% to 87.7% and from 39.7% to 70.3% in the YOA versus OA groups, respectively. In terms of occurrence, nine of the ten symptoms were the same for both groups (i.e., lack of energy, pain, worrying, feeling drowsy, dry mouth, feeling bloated, difficulty sleeping, lack of appetite, constipation). The symptoms that were unique were: feeling sad in the YOA group and numbness/tingling in hands/feet in the OA group.

**Table 3:** Rankings of the top ten symptoms based on occurrence, severity, and distress between the younger older adults and older adults

	Younger Older Adults			Older Adults		
Symptom Occurrence						
Rank	Symptom	%		Symptom	%	
1	Lack of energy	87.7		Lack of energy	70.3	
2	Pain	69.6		Feeling drowsy	59.1	
3	Worrying	69.0		Pain	54.7	
4	Feeling drowsy	59.6		Worrying	54.5	
5	Dry mouth	51.8		Dry mouth	52.3	
6	Feeling bloated	50.0		Difficulty sleeping	43.8	
7	Difficulty sleeping	48.3		Lack of appetite	42.2	
8	Feeling sad	48.3		Constipation	42.2	
9	Lack of appetite	46.6		Numbness/tingling in hands/feet	42.2	
10	Constipation	46.6		Feeling bloated	39.7	
Symptom Severity^a^						
Rank	Symptom	Mean	SD	Symptom	Mean	SD
1	Problem with sexual interest/activity	6.7	3.3	Constipation	5.3	2.5
2	Swelling of arms or legs	6.4	4.1	Hair loss	5.2	3.7
3	Changes in skin	6.2	3.1	Lack of appetite	4.9	2.4
4	Hair loss	6.0	3.5	Problem with sexual interest/activity	4.9	3.9
5	Feeling bloated	5.4	1.9	Changes in skin	4.9	2.9
6	Diarrhea	5.3	2.9	Pain	4.8	2.1
7	Problems with urination	5.1	2.8	I don’t look like myself	4.8	3.4
7	Sweats	5.1	3.0	Mouth sores	4.8	2.7
10	Vomiting	5.0	2.0	Problems with urination	4.6	4.0
10	Difficulty swallowing	5.0	2.2	Dry mouth	4.5	2.5
10	Shortness of breath	5.0	2.5	Feeling bloated	4.5	2.6
10	I don’t look like myself	5.0	2.9	Change in way food tastes	4.5	2.3
10	-------------------------------	---	---	Lack of energy	4.5	2.2
Symptom Distress^b^						
Rank	Symptom	Mean	SD	Symptom	Mean	SD
1	Changes in skin	7.3	2.4	Feeling nervous	5.2	2.9
2	Vomiting	6.5	0.7	Constipation	5.0	3.1
3	Problems with urination	5.4	2.8	Mouth sores	4.8	2.8
4	Swelling of arms or legs	5.0	5.2	Worrying	4.4	3.1
5	Difficulty swallowing	4.8	4.0	Feeling sad	4.2	2.6
6	Feeling nervous	4.6	2.3	Swelling of arms or legs	4.1	3.7
7	Shortness of breath	4.5	3.2	Feeling bloated	4.0	3.4
8	Worrying	4.5	2.7	Pain	4.0	3.0
9	Problem with sexual interest/activity	4.5	3.3	Lack of appetite	4.0	3.1
10	I don’t look like myself	4.4	4.1	I don’t look like myself	4.0	3.5
10	Constipation	4.4	3.4	Problems with urination	4.0	4.3

Abbreviation: SD, standard deviation.

^a^Symptom severity scores ranged from 0 (none) to 10 (intolerable).

^b^Symptom distress scores ranged from 0 (not at all distressing) to 10 (very distressing).

Severity and distress patterns indicate that the symptoms that were the most common, were not necessarily the ones that were the most severe or distressing. The most severe and distressing symptoms were more varied between the two groups than the occurrence of the symptoms. In the YOA, in terms of severity, twelve symptoms were included in Table 3, because four symptoms had the same score. In the OA, in terms of severity, thirteen symptoms were included in Table 3 because four of them had the same score. For severity, six symptoms were the same for both groups (i.e., problems with sexual interest/activity, changes in skin, hair loss, feeling bloated, problems with urination, I don’t look like myself). Symptoms that were unique to the YOA were: swelling of arms and legs, diarrhea, sweats, vomiting, difficulty swallowing, and shortness of breath. Symptoms that were unique to the OA were: constipation, lack of appetite, pain, mouth sores, dry mouth, changes in the way food tastes, and lack of energy. The severity scores for these symptoms ranged from 5.0 to 6.7 and from 4.5 to 5.3 in the YOA versus OA groups, respectively.

In the YOA, in terms of distress, eleven symptoms were included in Table 3, because two symptoms had the same score. In the OA, in terms of distress, eleven symptoms were included in Table 3 because five of them had the same score. For distress, six of the symptoms were the same in both groups (i.e., problems with urination, swelling of arms or legs, feeling nervous, worrying, I don’t look like myself, constipation). Symptoms that were unique for the YOA were: changes in skin, vomiting, difficulty swallowing, shortness of breath, and problems with sexual interest/activity. Symptoms that were unique to the OA were: mouth sores, feeling sad, feeling bloated, pain, and lack of appetite. The mean distress scores for the symptoms ranged from 4.4 to 7.3 and from 4.0 to 5.2 in the YOA versus OA groups, respectively.

Of note, in the OA, pain, feeling bloated, lack of appetite, and constipation were in the top ten symptoms for all three dimensions of the symptom experience (i.e., occurrence, severity, distress). In the YOA, no symptom was in the top ten for all three dimensions of the symptom experience.

## Discussion

This study is the first to perform a comprehensive evaluation of differences in multiple dimensions of the symptom experience between YOA and OA at initiation of chemotherapy. Of note, no differences were found in the total number of symptoms between the two age groups and overall symptom burden was similar. When significant differences were found, the YOA reported higher occurrence rates and higher severity and distress scores. Nine of the ten symptoms with highest occurrence rates were common for both age groups. For severity and distress, only half of the symptoms were common.

Similar to our previous study that compared older patients with low (< 2) and high (≥ 2) multimorbidity [25], as well as other studies [26–28], the symptoms with the highest occurrence rates were not always the most severe and/or the most distressing. Consistent with previous reports of older oncology patients [8, 10], while no age group differences were found, the mean number of symptoms in the YOA (11.0 ± 5.4) and OA (9.6 ± 5.6) equates with a moderate symptom burden at the initiation of chemotherapy [29]. This number of co-occurring symptoms suggests that both groups of older oncology patients have a moderate symptom burden prior to chemotherapy.

### Symptom occurrence

While nine of the ten symptoms with the highest occurrence rates were common to both age groups, it is interesting to note that in the previous study that evaluated symptom occurrence across four age groups (i.e., 60–64, 65–69, 70–74, ≥ 75), four of five symptoms were common across the age groups [8]. Three of these symptoms (i.e., lack of energy, pain, feeling drowsy) were common across our two age groups. In addition, these three symptoms had high occurrence rates in our older age groups. This finding is not surprising given that previous reports confirm the high occurrence rates for each of these symptoms [30–32]. In addition, these three symptoms are known to be three of five symptoms (i.e., fatigue, pain, disturbed sleep, drowsiness, lack of appetite) in a “sickness behavior” cluster identified in oncology patients [33]. Of note, all five symptoms in the sickness behavior cluster were among the top ten symptoms reported by both of our age groups.

It is interesting to note, while in the previous study [8] worrying was not among the top five symptoms (range from 24.4 to 40.8%), in our sample, 69.0% of YOA and 54.5% of OA reported this symptom. One possible explanation for this inconsistent finding is that symptoms were assessed at different times (i.e., at the initiation of versus during treatment). This hypothesis is supported by a previous study [5], that suggested that patients are more worried prior to treatment because they do not yet know what to expect.

In contrast to the previous report, where older patients reported a significantly lower occurrence rate for problems with sexual interest and higher rates for lack of appetite, dizziness, and swelling of arms or legs [8], no differences were found between the two age groups in the current study. These inconsistent findings may be related to how age was categorized as well as differences in cancer diagnoses and treatments. However, the OA in our study reported significantly lower occurrence rates for lack of energy and nausea. One possible explanation for why the YOA in our study reported high occurrence rates for lack of energy is that they were more likely to be employed. People in their 60s still perform tasks and have roles that create additional stress [34]. In addition, the higher occurrence rate for nausea may be due to the fact that younger patients report higher rates of nausea [35].

### Symptom severity

In terms of severity, only six symptoms were common between the YOA and OA. In addition, the severity scores for the top ten symptoms were in the moderate to severe range [36, 37] for the YOA (5.0 to 6.7) and OA (4.5 to 5.3). In contrast, the severity scores reported in the previous study of older adults [8] were in the slight to moderate range. One possible explanation for these inconsistent findings is that the symptoms were evaluated during treatment and these older oncology patients may have received more effective symptom management. These findings support the use of a multidimensional symptom assessment tool, like the MSAS, at initiation of chemotherapy.

Of note, in our OA, constipation and lack of appetite were the two unique symptoms among the top most severe and distressing. This finding is not surprising, given that the occurrence rate for constipation in our OA was 42.2% compared to rates of between 7.7% and 42.2% in the general population > 70 years of age [38]. In addition, constipation is known to increase with age in older community dwelling patients [39], and is reported to be one of the most troublesome symptoms in oncology patients [40]. Given that constipation can decrease oncology patients´ appetite and have detrimental effects on nutritional intake [40], clinicians need to assess for this symptom and initiate appropriate interventions.

For the YOA, vomiting was the unique symptom that had a significantly higher severity rating (i.e., 5.0 ± 2.0 versus 1.4 ± 1.9). While not in the top ten symptoms in terms of occurrence, vomiting was the second most distressing symptom for YOA and had a significantly higher distress score compared to the OA. While no differences in severity were found among the four age groups in the previous study [8], vomiting was one of the five most distressing symptoms in the youngest age group (60–64 years of age). It is not readily apparent why vomiting is ranked as more severe and distressing in the YOA at initiation of chemotherapy. However, our findings suggest that multiple dimensions of the symptom experience need to be assessed in older oncology patients.

In terms of difficulty swallowing, the YOA reported significantly higher severity ratings for this symptom (5.0 ± 2.2 versus 2.6 ± 1.7). This finding is consistent with a previous report of patients undergoing active cancer treatment [8], that found that as age increased, the severity of difficulty swallowing decreased in a linear fashion. Given that difficulty swallowing may result in malnutrition or aspiration pneumonia [41], clinicians need to assess for this symptom and initiate appropriate interventions.

### Symptom distress

Similar to severity, only six symptoms were common between the YOA and OA and their distress ratings were in the moderate to severe range for the YOA (4.4 to 7.3) and OA (4.0 to 5.2). The distress scores for the four age groups in the previous study [8] were slightly lower in the oldest age group (≥ 75 years of age). A potential explanation for both the higher distress and severity scores in our study is the occurrence of metastatic disease. While in the previous study [8], only 22–43% of the patients had metastatic disease, in our study this rate was between 75% and 81%. As noted in another study [32], the presence of metastatic disease is associated with increased symptom severity and distress. While metastatic disease is known to be a significant source of anxiety and distress [42], it is interesting to note that the older oncology patients in the previous study [8] reported lower distress scores for feeling nervous than the patients in our study.

The three common distressing symptoms not discussed previously were: problems with urination, swelling of arms or legs, and “I don’t look like myself”. Given that our patients had either gynecological or colorectal cancer and more than 50% of them had surgery prior to chemotherapy, it is not surprising that problems with urination was a common symptom [43, 44]. In addition, while the occurrence rates for swelling of arms or legs were relatively low (i.e., 8.8% and 19.7%), this symptom may be associated with underlying cardiovascular problems, given that 10.3% of the YOA and 15.9% of the OA reported heart disease and 24.1% and 45.5% reported high blood pressure. While the risk of cardiovascular disease in the cancer population is 800% higher than that general population [45], our findings suggest that patients with this symptom warrant an evaluation for cardiovascular disease [46].

“I don’t look like myself” was another common symptom associated with moderate levels of distress. As noted in three previous reports [47–49], a plausible explanation for this finding may be that the diagnosis of cancer and its treatments, as well as associated functional limitations, constitute to negative perceptions of body image and body satisfaction.

One of the four unique symptoms and the most distressing symptom in the YOA was changes in skin. In addition, changes in skin was significantly higher in the YOA adults compared to the OA (7.3 ± 2.4 versus 3.5 ± 3.6), respectively. Compared to the previous study with four age groups [8], changes in skin was not among the top five symptoms in any of the age groups and no differences were found across the groups. The lower level of distress associated with changes in skin in the OA may reflect a “response shift” in their perception of this symptom. A “response shift“ is defined as a change in the meaning of one’s self-evaluation as a result of changes in values or internal standards [50]. This hypothesis warrants confirmation in future studies.

While cancer and/or its treatments are known to effect patients´ sexual functioning [51], it is interesting to note that problems with sexual interest/activity was among the most severe symptoms in both age groups. However, higher levels of distress for this symptom was unique to the YOA. Our findings are somewhat consistent with a previous study [8], that found that patients ≥ 75 years of age, did not report problems with sexual interest/activity as the most severe or distressing symptom. A possible explanation for these findings is that sexual activity and a desire for sexual intimacy exhibits inter-individual variability regardless of age [52].

Several limitations warrant consideration. Because the sample was primarily women with gynecological cancer, our findings may not generalize to men and to older adults with other types of cancer. In addition, the patients had a MoCA score of ≥ 23, were predominantly well educated, and had metastatic disease which suggest that these findings may not generalize to all older oncology patients. In addition, while the total sample was relatively large, the two age groups were relatively small. Therefore, findings from this study warrant replication in a larger sample. Finally, because patients needed to have KPS score of ≥ 60 to participate in this study, older adults with a potentially higher symptom burden were not evaluated.

Consistent with previous studies of older patients [8, 10–12], few age-related differences were found in the various dimensions of the symptom experience. When some age differences were identified, the specific symptoms with differences in occurrence, severity, or distress varied among studies [8, 10–12]. In addition, consistent with two previous reports [8, 10], symptom occurrence rates were similar between our two age groups. For symptom severity ratings, over 50% of the highest rated symptoms was unique in our age groups. For distress, the unique symptoms varied across studies (i.e., 100% [8], 50% in our study, and 20% [10]).

## Conclusions

Taken together, these findings suggest that for the most prevalent, severe, and distressing symptoms in older oncology patients, age differences may be related to a variety of factors (i.e., cancer diagnosis, timing of the assessment, presence of metastatic disease). Oncology clinicians need to perform routine assessments of symptom severity and distress in older patients prior to treatment and initiate targeted symptom management interventions. This comprehensive evaluation of symptoms should be performed throughout treatment. Longitudinal studies are needed to evaluate for changes in older oncology patients´ symptom burden across their disease and treatment trajectories. Given that variability exists in the symptom experience of older oncology patients across heterogeneous types of cancer, future studies should evaluate for age-related differences within specific types of cancer.

## Data Availability

Due to restrictions from the Regional Committee for Medical and Research Ethics, data for this study are not available, but are available from the corresponding author on reasonable request.

## List of abbreviations

ESAS: Edmonton Symptom Assessment Scale
KPS: Karnofsky Performance Status
MoCA: Montreal Cognitive Assessment
MSAS: Memorial Symptom Assessment Scale
OA: Older adults
SCQ-16: Self-Administered Comorbidity Questionnaire
SPSS: Statistical Package for the Social Sciences
WHO: World Health Organization
YOA: Younger older adults
